# Joint developmental trajectories and temporal precedence of physical function decline and cognitive deterioration: A longitudinal population-based study

**DOI:** 10.3389/fpsyg.2022.933886

**Published:** 2022-10-12

**Authors:** Xiao Wei, Heng Liu, Li Yang, Zihan Gao, Jinke Kuang, Kexin Zhou, Mengfan Xu

**Affiliations:** School of Nursing, Qingdao University, Qingdao, China

**Keywords:** cognitive function, physical function, aging, latent growth model, autoregressive cross-lagged model

## Abstract

**Objectives:**

Previous studies primarily explored the unidirectional impact of cognition on physical function. However, the interplay between physical function and cognition and the temporal precedence in their predictive relationships have not been elucidated. We explored the bidirectional mechanism between physical function and cognition in a longitudinal dataset.

**Materials and methods:**

A total of 1,365 participants in the Chinese Longitudinal Healthy Longevity Survey assessed physical function and cognition in 2011 (T1), 2014 (T2), and 2018 (T3) by the Katz scale and the Chinese version of the Mini-Mental State Examination scale, respectively. Changes in the trajectories of physical function and cognition were examined using the latent growth model. The correlational and reciprocal relationships between physical function and cognition were examined using the parallel process latent growth model and autoregressive cross-lagged (ARCL) models.

**Results:**

Cognition and physical function decreased by an average of 0.096 and 0.017 points per year, respectively. Higher physical function was associated with better cognition at baseline (*r* = 0.237, *p* < 0.05), and longitudinal changes in physical function and cognition were positively correlated (*r* = 0.756, *p* < 0.05). ARCL analysis indicated that physical function at T1 positively predicted T2 cognitive function. However, this predictive relationship reversed between T2 and T3, whereby cognitive function at T2 predicted physical function at T3.

**Conclusion:**

Both physical function and cognition declined over time. Early identification and intervention in physical dysfunction among older adults could be critical to prevent further cognitive impairment and maintain functional independence. Hence, regular functional assessment and individualized care plans are required to achieve healthy aging.

## Introduction

The number of adults aged ≥ 60 years is expected to reach 2 billion by 2050, representing 22% of the entire population, almost double the 12% reported in 2015 (World Health Organization, 2018). Meeting the health needs of an aging population of this size poses a serious challenge to global health systems. It is accepted that prevention is more important than treatment in achieving healthy aging (Nivestam et al., 2020). Functional decline is a largely preventable feature of aging and is defined as a progressive weakening of functional autonomy, comprising mainly cognitive deterioration and decline in physical function (PF), impairing the ability of older adults to live safely and independently (Grimmer et al., 2013). Recognizing the characteristics of cognitive function (CF) and PF decline is crucial for supporting healthy aging and maintaining the quality of life of older adults.

Cognitive deterioration is placed on a continuum, including normal aging, mild cognitive impairment, and severe cognitive impairment (dementia) (Chertkow et al., 2007) that are accompanied by different PF states, especially in the areas of feeding, dressing, toileting, and bathing (Ebly et al., 1995). Physical function in the dementia group was lower than that in the mild cognitive impairment group, specifically, regarding grooming, bathing, and bowel control domains (Lee et al., 2019). Cognitive impairment has been proven to be a risk factor for PF decline among older adults (Arias-Merino et al., 2012; Ha and Kim, 2014). Several studies on the trajectories of functional capacity decline have revealed that participants with worse CF at baseline exhibited larger PF decline (Dodge et al., 2005; Helvik et al., 2015), and older adults with lower levels of cognition are more likely to decline in functional capacity (Menezes et al., 2021).

Relatively few studies have focused on the effects of PF on cognition. However, available research has begun to propose that cognitive deterioration and PF decline may mutually influence and reinforce each other’s development (Black and Rush, 2002). One study reported that the onset of functional disability accelerated CF decline that progressed faster in individuals with more severe functional disabilities (Rajan et al., 2013). This echoes the findings of another study that the prevalence of mild cognitive impairment in individuals with disabilities is higher than that in persons without disabilities (Chang et al., 2017).

In summary, many of the existing studies have focused on the unidirectional relationship between CF and PF. Meanwhile, evidence from cross-sectional studies has limited utility when considering temporal associations between variables. Moreover, existing longitudinal studies have focused more on the contributions of longitudinal changes in cognition to longitudinal changes in PF (Dodge et al., 2006; Cahn-Weiner et al., 2007), leading to the possibility that existing studies overlooked the impact on cognitive function of PF decline due to various causes, such as falls and fractures, when cognitive function is not impaired. Clarifying whether PF or cognitive ability changes first during the aging process and the subsequent impact on each other would better guide functional screening and interventions for older adults. Hence, longitudinal studies exploring the reciprocal relationship between PF and cognition are required.

In addition, previous studies have shown that changes in cognitive function varied significantly by sex, age, educational level, income, living arrangements, psychological status, social participation, and age-related health conditions such as hearing function and the number of chronic diseases (Zhang et al., 2019; Chen and Zhou, 2020). However, the association between lifestyle factors, such as smoking and alcohol consumption, and cognitive impairment is inconsistent across studies. Some studies reported that smoking status and excessive alcohol consumption are associated with cognitive decline in older adults (Hagger-Johnson et al., 2013), whereas other studies have disproven this association (Hou et al., 2018; Zhang et al., 2019). Similarly, studies demonstrated that sex, age, education level, depressive symptoms, and age-related health conditions such as hearing function and the number of chronic conditions are associated with PF decline in older adults (Diaz-Venegas et al., 2016; Chen and Zhou, 2020). Therefore, the current study includes all these variables as a covariate to identify protective or risk factors for CF and PF in older adults.

Hence, this study aimed to explore the longitudinal correlational and reciprocal relationships between PF and CF by utilizing large-scale data from the older adult Chinese population and advanced statistical methods, including the latent growth model (LGM) and an autoregressive cross-lagged (ARCL) model.

## Materials and methods

### Participants and settings

The data were obtained from the Chinese Longitudinal Healthy Longevity Survey (CLHLS), a longitudinal study investigating the health status of older adults in China, conducted by the Center for Research on Healthy Aging and Development at Peking University, which obtained data from eight face-to-face interviews with the cohort since 1998, utilizing internationally compatible questionnaires. Survivors were reinterviewed at each follow-up visit, and those deceased were substituted with new participants (Yang et al., 2016). Of 31 provinces in China, 23 were randomly selected, accounting for approximately half of the urban areas in each province (Qiu et al., 2020). The age of the respondents who voluntarily agreed to participate in the study covered the middle age group (34–64 years), the lower age group (65–80 years), and the higher age group (80 years and above). Individuals were excluded if they died, were lost to follow-up, or failed to respond to the items comprising the outcome variable (Ni et al., 2020). More detailed data information is available at https://doi.org/10.18170/DVN/WBO7LK.

We used data from three waves of the CLHLS: 2011 (T1), 2014 (T2), and 2018 (T3) as a result of follow-up loss and natural death of subjects, and the first measurement of health information including hearing function in 2011. The two inclusion criteria were (1) completion of three surveys and (2) no missing PF and CF data. There were 9,765 subjects in 2011. By 2018, 6,862 subjects were lost to follow-up or had died during the follow-up period. After excluding a further 1,538 subjects with missing PF and CF data, we ultimately analyzed 1,365 individuals who responded to the survey from 2011 to 2018.

We compared the baseline characteristics of included and excluded participants (see Supplementary Table 1). The results revealed no differences between included and excluded participants regarding income, living arrangements, and number of chronic conditions. Predictably, and consistent with previous studies (Yu et al., 2020; Li et al., 2021), these excluded participants tended to be older, female, with lower levels of education, who smoked less, drank less alcohol, and performed less well in terms of mental status, social engagement, and hearing function, but did not differ from included participants in terms of income, living arrangements, and number of chronic conditions.

### Cognitive function

Cognitive function in the CLHLS was tested using the Chinese version of the Mini-Mental State Examination (C-MMSE), which comprises six subscales with 24 items: five orienting, three registering, one naming, five noticing and counting, three recalling, and seven verbal items. The total C-MMSE rating ranged from 0 to 30 points, with larger scores indicating stronger cognition. The C-MMSE is a well-established instrument for CF assessment that has been verified in previous studies (Yang et al., 2016; Zhang et al., 2019; Qiu et al., 2020).

### Physical function

Physical function was evaluated by sum scores (ranging from 6 to 18 points) in six components of daily living: bathing, dressing, grooming, indoor transferring, toileting, and feeding according to the Katz scale (Katz et al., 1963). Older adults were scored as 1 (totally dependent on others), 2 (partially independent), or 3 (completely independent) points, depending on their ability to complete these actions without assistance, with larger scores indicating better daily living ability.

### Covariates

The following covariates were evaluated in this study: (1) demographic characteristics, including age, sex (1 = man, 2 = woman), years of education, living arrangement (1 = living with family members, 0 = not living with family members), and income; (2) lifestyle factors, including smoking (1 = yes, 0 = no) and drinking (1 = yes, 0 = no); (3) social engagements, including doing housework, working in the garden, reading newspapers/books, raising domestic poultry/pets, playing poker/mahjong, watching television or listening to the radio, and participating in social activities. Respondents were asked about the frequency of participation in each activity: 5 = almost every day, 4 = at least once a week, 3 = at least once a month, 2 = sometimes, and 1 = never; (4) mental status, representing the extent of mental wellbeing, rated by the sum of seven questions scored on a scale from 7 to 35 points, including being optimistic, maintaining tidiness, not feeling afraid or restless, not feeling alone, making own decisions, not feeling useless with age, and being happier than in youth, with better scores indicating a more positive mental state; (5) health status, including hearing function and the numbers of chronic diseases, where the hearing function was assessed based on the interviewee’s ability to hear the interviewer’s question, with four possible responses: 4 = yes, without hearing aid; 3 = yes, but needs hearing aid; 2 = partly, despite hearing aid; 1 = no. Chronic diseases included hypertension, diabetes, heart disease, stroke, bronchitis, tuberculosis, cataracts, glaucoma, cancer, gastric or duodenal ulcer, Parkinson’s disease, bedsore, arthritis, and dementia. Sex, education, and income levels were time-invariant covariates, while the other variables were time-varying covariates.

### Data analysis

The participants’ characteristics, including CF scores, PF scores, and covariate distributions or scores, were presented as descriptive statistics and analyzed using SPSS 25.0 (IBM Corp., Armonk, NY, United States). Missing covariate data were substituted using multiple imputations based on Bayesian methods in SPSS 25.0.

### Latent growth model

The LGM allows analysis of repeatedly measured temporal trajectories of a variable by establishing random intercepts and slopes (Sha et al., 2018), thereby describing individual trajectories and capturing individual differences in trajectory variation over time (Wang et al., 2017). See the Supplementary Data Sheet 1 for the specific rationale. Therefore, LGM analysis was performed to examine the changing trajectories of PF and CF. The change in trajectory was represented by two latent variables: a potential intercept growth factor, which reflects the initial state of variables, and a potential slope growth factor, which reflects the changing rate of variables. By considering the three waves of data, the trajectories of the variables were modeled using the specified linear LGM. The loading of the intercept factor was set for each measurement at a constant value of 1.0. For the slope factor, we fixed loadings sequentially at 0, 3.0, and 7.0, based on the survey time point.

**FIGURE 1:**
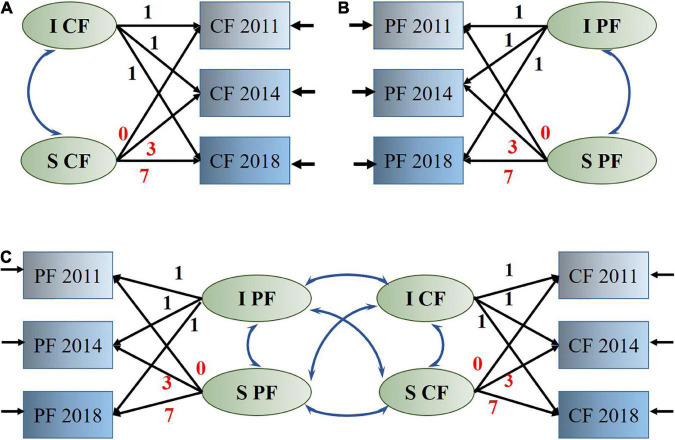
Latent growth model. **(A)** Measurement of latent growth model for cognitive function. **(B)** Measurement of latent growth model for physical function. **(C)** Structural latent growth model to assess the relationships between changes in physical function and cognitive function. PF, physical function; CF, cognitive function; I, intercept; S, linear slope.

Two unconditional LGMs were first modeled to mirror the growing trajectories of PF and CF without predictor variables (Figures 1A,B). The parallel process LGM (PP-LGM) enabled the estimation of interrelationships between growth factors (intercept and slope) by building a hypothetical model of two variables in parallel processes (Ni et al., 2020). Unconditional (without covariates or predictors, Figure 1C) and conditional (with covariates or predictors added) PP-LGMs were established to examine whether the growth parameters of one trajectory were related to those of the other.

### Autoregressive cross-lagged model

The ARCL model, comprising an autoregressive component and a cross-lagged component, adjusts for the stability of all variables over time and simultaneously explores the interaction of the two variables over time (Han and Kim, 2020). Thus, it is often applied to deduce temporal precedence of predictive relationships among variables in longitudinal studies. The cross-lagged parameters are typically interpreted as the between-person effect of X_*time1*_ on Y_*time2*_, controlling for Y_*time1*_ and vice versa (Gueron-Sela and Gordon-Hacker, 2020). Therefore, the ARCL model helped to distinguish between the effects of PF on CF and the effects of CF on PF in the current study, reflecting the reciprocal relationship between the two variables over an extended period.

All LGM and ARCL model analyses were conducted using Mplus version 7.0 (Muthén & Muthén, Los Angeles, CA, United States). The following metrics were used to evaluate the model fit: chi-square (χ^2^) statistic, 1 < χ^2^/degrees of freedom (df) < 3, comparative fit index (CFI) of > 0.90, preferably of > 0.95; root mean square error of approximation (RMSEA) of < 0.08; 90% confidence interval (CI) ≤ 0.08, preferably of < 0.06 (Melka et al., 2011); and standardized root mean square residual (SRMR) of ≤ 0.08 (Brown and Weisman de Mamani, 2018).

## Results

The respondents’ characteristics are presented in Table 1. In the baseline survey, 54.9% of the participants were men, and 81.8% lived with their families. Most participants were non-smokers and non-drinkers; the corresponding proportions of participants increased during the follow-up period. During the 7-year period, the scores of covariates reflecting healthy status, including mental status, social engagements, and hearing function, declined with increasing age and the number of chronic diseases. The mean cognitive and physical functioning scores at each time point also decreased over time.

**TABLE 1:** Characteristics of the study participants.

Variable categories	Variables	2011	2014	2018
Time-invariant covariates	**Gender, %**			
	Female	45.1	—	—
	Male	54.9	—	—
	Years of education, Mean ± SD	3.52 ± 3.83	—	—
	Incomes, Mean ± SD	4.12 ± 0.52	—	—
Time-varying covariates	**Living arrangement, %**			
	With family members	81.8	79.4	80.4
	Not living with family members	18.2	20.6	19.6
	**Smoking, %**			
	Yes	22.8	21.2	19
	No	77.2	78.8	81
	**Drinking, %**			
	Yes	22.3	19.8	18.7
	No	77.7	80.2	81.3
	**Age (years), median, [IQR]**	74 [70–79]	77 [73–82]	81 [77–86]
	Numbers of chronic diseases, Mean ± SD	1.05 ± 1.06	1.04 ± 1.16	1.14 ± 1.14
	Mental status, Mean ± SD	27.31 ± 3.73	27.03 ± 3.73	26.67 ± 3.72
	Social engagements, Mean ± SD	18.34 ± 4.84	18.20 ± 5.07	15.98 ± 4.90
	Hearing function, Mean ± SD	2.94 ± 0.31	2.92 ± 0.35	2.85 ± 0.50
Main variables	ADL scores, Mean ± SD	17.93 ± 0.41	17.92 ± 0.48	17.79 ± 0.90
	Cognitive scores, Mean ± SD	28.27 ± 2.22	28.15 ± 2.49	27.59 ± 3.12

Person *N* = 1,365. IQR, inter-quartile range.

### Measurement model

In the LGM, the two trajectories of CF and PF change were described by a linear model with satisfactory fit indices (Table 2). Based on the mean intercept representing the baseline level, the initial scores for PF and CF were estimated to be 17.942 and 28.323 points, respectively. Based on the mean slope indicating the direction and magnitude of the trajectory change, the mean annual reductions in PF and CF scores were separately estimated at 0.017 and 0.096 points, respectively. Roughly, these results are consistent with the descriptive statistics on CF and PF as shown in Table 1, indicating that this LGM fits well to the data.

**TABLE 2:** Results of univariate latent growth model analyses.

Model	Intercept				Slope				Goodness-of-fit indices
			
	Mean	*P*	Variance	*P*	Mean	*P*	Variance	*P*	
Physical function	17.942	< 0.001	0.051	< 0.001	−0.017	< 0.001	0.003	0.002	χ^2^(1) = 8.810, *P* = 0.003, χ^2^/df = 8.810; CFI = 0.924; SRMR = 0.034; RMSEA = 0.076 (0.036–0.0125)
Cognitive function	28.323	< 0.001	1.600	< 0.001	−0.096	< 0.001	0.050	0.002	χ^2^(1) = 5.805, *P* = 0.016, χ^2^/df = 5.805; CFI = 0.984; SRMR = 0.02; RMSEA = 0.059 (0.020–0.110)

χ^2^, chi-square; df, degrees of freedom; CFI, comparative fit index; SRMR, standardized root mean square residual; RMSEA, root mean square error of approximation.

### Structural model

#### Unconditional parallel process-latent growth model modeling

The unconditional PP-LGM used to test the correlation between PF decline trajectory with the trajectory of CF deterioration showed a better model fit (Table 3). The PF intercept was significantly associated with the CF intercept in 2011 (*r* = 0.237, *p* < 0.05), which suggested that participants with better PF had higher cognitive scores at baseline. The PF slope was significantly associated with the CF slope (*r* = 0.756, *p* < 0.05), indicating that subjects with a greater rate of PF decline experienced greater decreases in CF over time.

**TABLE 3:** Standardized coefficients for parallel process LGM.

Models	Parameters	Coefficients	*P*	Goodness-of-fit indices
Unconditional model	PF intercept ↔ CF intercept	0.237*	0.005	χ^2^(7) = 39.297, *P* < 0.001, χ^2^/df = 5.614; CFI = 0.932; SRMR = 0.074; RMSEA = 0.058 (0.041–0.076)
	PF intercept ↔ CF slope	−0.139	0.217	
	PF slope ↔ CF intercept	−0.028	0.790	
	PF slope ↔ CF slope	0.756*	< 0.001	
Conditional model ^a^	PF intercept ↔ CF intercept	0.169	0.057	χ^2^(109) = 181.182, *P* < 0.001, χ^2^/df = 1.662; CFI = 0.941; SRMR = 0.014; RMSEA = 0.022 (0.016–0.028)
	PF intercept ↔ CF slope	−0.118	0.226	
	PF slope ↔ CF intercept	−0.123	0.312	
	PF slope ↔ CF slope	0.464*	0.003	

LGM, latent growth model; PF, physical function; CF, cognitive function; χ^2^, chi-square; df, degrees of freedom; CFI, comparative fit index; SRMR, standardized root mean square residual; RMSEA, root mean square error of approximation. ^a^The covariates were age, sex, marital status, education, residential areas, living arrangement, income, smoking, alcohol drinking, social engagement, number of chronic diseases, and hearing function. *P < 0.05.

#### Conditional parallel process-latent growth model modeling

Compared with the unconditional PP-LGM, the conditional PP-LGM that controlled for predictors yielded better fit indices, but the results differed (Table 3). The correlation between initial PF levels and baseline CF scores was non-significant (*r* = 0.169, *p* > 0.05). Only the correlation between PF slope and CF slope remained significant, and the standardized coefficients were attenuated (*r* = 0.464, *p* < 0.05).

The variances of intercepts and slopes for PF and CF scores were significant (*p* < 0.05, Table 2), indicating strong individual differences between the trajectories of PF and CF. Therefore, time-invariant and time-varying covariates were included in the PP-LGM to determine whether individual characteristics were predictive of bivariate trajectories of CF and PF.

Sex, living arrangement, drinking status, and the number of chronic diseases were negatively associated with initial CF scores. Participants with higher education or lower incomes had a greater CF decline rate. Meanwhile, mental status, social engagement, and hearing function were positively related to CF scores at all three time points. However, the hearing function was positively correlated with PF in the last two waves. Living with family members and more chronic diseases was associated with lower PF scores in the third wave (Table 4).

**TABLE 4:** Standardized coefficients for covariates in the conditional parallel process latent growth model.

Correlations	β	*P*	Correlations	β	*P*
**Time-invariant covariates**					
Gender →CF Intercept	−0.206*	< 0.001	Gender →PF Intercept	0.019	0.726
Gender →CF Slope	–0.022	0.730	Gender →PF Slope	0.009	0.906
Years of education →CF Intercept	0.202*	< 0.001	Years of education →PF Intercept	–0.075	0.170
Years of education →CF Slope	0.140*	0.031	Years of education →PF Slope	–0.068	0.373
Incomes →CF Intercept	0.210*	< 0.001	Incomes →PF Intercept	–0.009	0.859
Incomes →CF Slope	−0.179*	0.004	Incomes →PF Slope	–0.105	0.149
**Time-varying covariates**					
Age1→CF1	−0.140*	< 0.001	Age1→PF1	−0.058*	0.037
Age2→CF2	−0.156*	< 0.001	Age2→PF2	−0.104*	< 0.001
Age3→CF3	−0.157*	< 0.001	Age3→PF3	−0.098*	< 0.001
Living arrangement1→CF1	−0.068*	0.010	Living arrangement1→PF1	–0.054	0.055
Living arrangement2→CF2	0.015	0.538	Living arrangement2→PF2	–0.033	0.212
Living arrangement3→CF3	0.006	0.790	Living arrangement3→PF3	−0.054*	0.040
Smoke1→CF1	–0.020	0.456	Smoke1→PF1	0.013	0.652
Smoke2→CF2	–0.023	0.375	Smoke2→PF2	–0.015	0.582
Smoke3→CF3	–0.025	0.326	Smoke3→PF3	–0.013	0.646
Drink1→CF1	−0.053*	0.047	Drink1→PF1	0.032	0.262
Drink2→CF2	0.002	0.938	Drink2→PF2	0.036	0.186
Drink3→CF3	–0.002	0.931	Drink3→PF3	0.035	0.202
Numbers of chronic diseases1→CF1	−0.055*	0.030	Numbers of chronic diseases1→PF1	–0.051	0.061
Numbers of chronic diseases2→CF2	–0.035	0.152	Numbers of chronic diseases2→PF2	–0.050	0.055
Numbers of chronic diseases3→CF3	−0.052*	0.029	Numbers of chronic diseases3→PF3	−0.085*	0.001
Mental status1→CF1	0.118*	< 0.001	Mental status1→PF1	0.039	0.163
Mental status2→CF2	0.096*	< 0.001	Mental status2→PF2	0.030	0.242
Mental status3→CF3	0.101*	< 0.001	Mental status3→PF3	0.046	0.087
Social engagements1→CF1	0.084*	0.001	Social engagements1→PF1	0.100*	< 0.001
Social engagements2→CF2	0.071*	0.005	Social engagements2→PF2	0.086*	0.001
Social engagements3→CF3	0.160*	< 0.001	Social engagements3→PF3	0.180*	< 0.001
Hearing function1→CF1	0.088*	< 0.001	Hearing function1→PF1	0.034	0.190
Hearing function2→CF2	0.145*	< 0.001	Hearing function2→PF2	0.118*	< 0.001
Hearing function3→CF3	0.176*	< 0.001	Hearing function3→PF3	0.078*	0.003

PF, physical function; CF, cognitive function; 1, year 2011; 2, year 2014; 3, year 2018. *P < 0.05.

### Bidirectional associations between physical function and cognition

**FIGURE 2:**
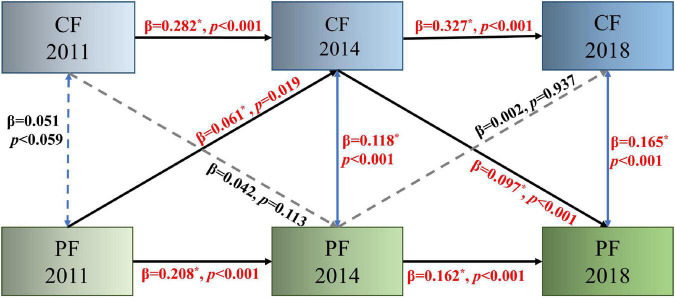
Autoregressive cross-lagged model assessing bidirectional relationships between physical function and cognition over 3 years. Solid lines indicate significant associations, and dashed lines indicate non-significant associations. PF, physical function; CF, cognitive function, *p < 0.05.

We inspected the bidirectional association between PF and CF at three time points using the ARCL model. The model fits the data well [χ^2^/(4) = 33.78, *p* < 0.001, CFI = 0.937, RMSEA = 0.074, RMSEA 90% CI (0.052, 0.098), and SRMR = 0.026]. The significant autoregressive paths indicate the stability of PF and CF over time. PF in the first wave had a statistically significant effect on cognition in the second wave (β = 0.061, *p* = 0.019) and cognition in the second wave on PF in the third wave (β = 0.097, *p* < 0.001) (Figure 2). Compared with the result which controls for covariates (see Supplementary Figure 1), the results show the same predictive chain that between T1 and T2, PF positively predicted CF. However, from T2 to T3, the opposite effect was observed, whereby CF positively predicted PF.

## Discussion

The present study investigated the trajectories of PF performance and CF scores and the longitudinal relationships between PF decline and cognitive deterioration in the Chinese older population based on longitudinal data collected over 7 years. The LGM revealed a declining trend in both CF and PF scores with a low change rate, consistent with a previous study that reported a minor decline in PF and cognition, with average annual decline rates of 0.03 (Diaz-Venegas and Wong, 2016) and 0.07 (Ku et al., 2012), respectively. The finding can be explained by the “benefits of success” concept (Fries, 1980); in other words, persons are living longer (success) and in better health at older ages than previously (benefits). The cognition and PF of older adults have benefited from these advances, with lower rates of functional disability, more effective disease treatment and healthier lifestyles, reduced disability rates for some major chronic diseases, such as stroke and cardiovascular disease, and improved living standards due to China’s rapid socio-economic development (Zeng et al., 2017). Overall, the decrease in PF and CF is insidious. Regular assessments of CF and PF are essential to achieving good outcomes and should ideally be included in routine medical examinations of middle-aged and older individuals.

The correlational relationship between CF and PF was explored using PP-LGM. Individuals with lower PF levels at baseline showed lower CF scores. However, the weak correlation (Cohen, 1992) between PF and CF at baseline was weakened to insignificance by the inclusion of control variables as competing explanatory variables (Lee Ray, 2016) during the initial CF, and PF of the study subjects was less impaired. Both initial cognitive functioning and PF performance were shown to be primarily influenced by age and social engagements in this study. Consistent with previous research, social engagement helps protect against functional decline in PF and CF among older adults, namely more social engagement is associated with better cognitive performance and less PF decline. The cognitive reserve, stress, and vascular hypothesis proposes that social activity can increase cognitive reserve to selectively improve CF and delay CF decline by increasing social contact with people to maintain a positive emotional state or reduce stress levels and consequently reduce the risk of cardiovascular disease (Fratiglioni et al., 2004). Other studies have reported the protective effect of social participation on PF (Ma et al., 2020; Dos Santos and Alberto Gobbo, 2021), even if older adults only do chores around the house that require strength and limb exercises in certain situations, such as carrying a bucket of water or sweeping, sufficiently to contribute to the maintenance of PF. Health policy initiatives should therefore encourage older adults to engage in social activities to delay their functional decline, including the provision of community activity centers for older adults.

However, the decreased rate of PF is positively correlated with the rate of CF deterioration, independently of adjustments for covariates, indicating that accelerated decline in function on either side triggers accelerated functional decline on the other side. It means the accelerated PF decline could trigger an accelerated decline in cognitive function and vice versa, which corroborates that PF and CF reciprocally influence each other’s development (Black and Rush, 2002) and may form a self-perpetuating malignant cycle in the absence of appropriate interventions. This finding deserves more attention from clinicians. Focusing on disability prevention and PF exercise could reduce the occurrence of cognitive decline. Equally, attention to the prevention and treatment of cognitive impairment will potentially prevent functional disability. Thus, attention to risk factors for cognitive or physical decline, such as specific chronic health conditions and mental status, would be helpful in preventing functional decline in older adults.

We further used the ARCL model to detect the sequence of predicted relationships between PF decline and CF deterioration. Consistent with a previous study (Farias et al., 2013) showing a gradual decline of daily functioning in individuals with normal CF and an accelerated decline as MCI progresses toward dementia, our results support that PF decline precedes CF decline and subsequently induces more PF decline. It could be explained by two potential mechanisms. First, neurofibrillary tangles in the substantia nigra related to gait may represent preclinical cognitive impairment in the brain pathology (Schneider et al., 2006). Second, stroke or diabetes can induce an initial reduction in PF, and disease-related vascular problems may subsequently exacerbate CF decline (Fauth et al., 2013).

The specific predictive chronology of functional decline identified in this study suggests that the health goals of older adults at different levels of functioning are distinct and that health management programs should focus on effectively preventing and delaying PF decline. Focusing on the etiology of unintentional injuries, such as falls, fractures, and cardiovascular disease, may help prevent or enable early intervention in PF decline, thereby helping to maintain a satisfactory quality of life.

This longitudinal study was based on a large national sample from the CLHLS database. Data quality was ensured by implementing strict quality control measures at each stage of the CLHLS survey. The use of LGM and ARCL analyses helped to identify the joint trajectory characteristics and sequential relationships between PF and CF decline, helping to determine the stage of functional decline among older adults, to select the timing of intervention, and to develop targeted health management programs that aim to delay functional decline and achieve healthy aging. Moreover, this study identified factors affecting both PF and cognition, which helped elucidate the association between PF and CF.

The present study has some limitations. Although the data quality was assured by stringent quality control at each stage of the CLHLS, the long duration of follow-up yielded missing data. The study performed multiple interpolations of data with missing confounding variables to achieve the best possible use of the available data. Furthermore, retained cohort members were younger and had better mental status and social engagement in general than excluded individuals, which may lead to the study being underpowered and biased toward the healthier segment of the aging population. Since we used a nationally representative database, confirmation bias could be limited to an extent. We are reminded to expand the sample in future studies by adding older individuals with different mental statuses and social engagement levels as subjects to improve the generalizability of this study’s findings. In addition, the research items, including PF assessment, social engagement, and mental status, were based on self-reported data, which may have been affected by recall and measurement biases. Nevertheless, the face-to-face interview method allowed investigators to get a more accurate picture of overall respondent profiles, which ensured the response quality to some extent. Furthermore, although this study revealed declines in cognitive and physical function among older adults with three measurements over a 7-year period, the two intervals may not be long enough to detect significant functional decline and to examine the non-linear relationship between PF and cognition. Therefore, future studies should consider selecting objective measurement instruments and extending the follow-up period to explore the long-term dynamic relationship between PF and cognition by constructing non-linear LGM.

## Conclusion

This longitudinal study examined the temporal relationship between PF and CF in Chinese older adults, using survey data collected at three time points over 7 years. The results revealed that a decrease in PF and CF was characterized as implicit, with accelerated decline in one variable causing a faster decrease in the other variable. There is a clear prediction priority in the time between PF decrement and cognitive deterioration, whereby initial PF scores predicted CF, and CF predicted subsequent PF. Early identification and intervention in physical dysfunction among older adults would be critical to prevent further cognitive impairment and maintain functional independence. Regular functional assessment and individualized care plans are required to achieve healthy aging.

## Data availability statement

The data used were obtained from the public database of the Chinese Longitudinal Healthy Longevity Survey: https://opendata.pku.edu.cn/dataset.xhtml?persistentId=doi:10.18170/DVN/WBO7LK.

## Author contributions

XW and HL were responsible for the study conception, design, and drafting of the manuscript. ZG, JK, KZ, and MX collated data and conducted data analyses. LY was responsible for supervision. All authors made critical revisions to the manuscript.
