# Supplementary material for: Joint developmental trajectories and temporal precedence of physical function decline and cognitive deterioration: A longitudinal population-based study
Source: Front Psychol. 2022 Oct 12;13:933886. doi: 10.3389/fpsyg.2022.933886 (PMC9597508; doi:10.3389/fpsyg.2022.933886)
Supplement: Supplementary file 1 [file Table_1.docx]

Table S1. Comparison of baseline characteristics between included and excluded participants

| **Participants’ Characteristics  at baseline** | **Included** | **Excluded** | **t/Z** | ***P*-Value** |
| --- | --- | --- | --- | --- |
|  | **(N=1365)** | **(N=8400)** |  |  |
| **Median Age at baseline [IQR]** | 74 [70,79] | 88 [79,96] | -37.06 | <.0001 |
| **Gender** |  |  | -7.873 | <.0001 |
| Male (%) | 749 (54.9) | 3649 (43.4) |  |  |
| Female (%) | 616 (45.1) | 4751 (56.6) |  |  |
| **Years of education, Mean ± SD** | 3.52± 3.83 | 2.10±3.41 | -12.92 | <.0001 |
| Miss (%) | 1 (0.1) | 40 (0.5) |  |  |
| **Income** | 4.12±0.54 | 4.13±0.56 | 0.53 | 0.60 |
| Miss (%) | 119 (8.7) | 951 (11.3) |  |  |
| **Living arrangement** |  |  | -0.731 | 0.47 |
| With family members (%) | 1103 (80.8) | 6701 (79.8) |  |  |
| Not living with family members (%) | 247 (18.1) | 1586 (18.9) |  |  |
| Miss (%) | 15 (1.1) | 113 (1.3) |  |  |
| **Smoking** |  |  | -5.111 | <.0001 |
| Yes (%) | 311 (22.8) | 1422 (16.9) |  |  |
| No (%) | 1049 (76.8) | 6879 (81.9) |  |  |
| Miss (%) | 5 (0.4) | 99 (1.2) |  |  |
| **Drinking** |  |  | -5.373 | <.0001 |
| Yes (%) | 299 (21.9) | 1341 (16.0) |  |  |
| No (%) | 1050 (76.9) | 6922 (82.4) |  |  |
| Miss (%) | 16 (1.2) | 137 (1.6) |  |  |
| **Numbers of chronic diseases, Mean ± SD** | 1.07±1.22 | 1.12±1.25 | 1.04 | 0.30 |
| Miss | 430 (31.5) | 2162 (25.7) |  |  |
| **Mental status, Mean ± SD** | 27.33± 3.77 | 26.20±4.01 | -0.9 | <.0001 |
| Miss | 53 (3.9) | 1689 (20.1) |  |  |
| **Social engagements, Mean ± SD** | 18.34± 4.85 | 16.67± 6.66 | -11.05 | <.0001 |
| Miss | 4 (0.3) | 96 (1.1) |  |  |
| **Hearing function, Mean ± SD** | 2.95± 0.31 | 2.27±1.11 | -46.08 | <.0001 |
| Miss | 1 (0.1) | 64 (0.8) |  |  |

*Notes.* IQR, Inter-quartile range.
