# Supplementary material for: Joint developmental trajectories and temporal precedence of physical function decline and cognitive deterioration: A longitudinal population-based study
Source: Front Psychol. 2022 Oct 12;13:933886. doi: 10.3389/fpsyg.2022.933886 (PMC9597508; doi:10.3389/fpsyg.2022.933886)
Supplement: Supplementary file 2 [file Data_Sheet_1.docx]

Rationale of the latent growth model(Mengcheng & Xiangyang, 2018) (Grimm & Ram, 2018)

In a standard linear LGM, each individual growth trajectory with respect to a variable is modelled by the following equation:

$y_{it}=a_{i}+\beta_{i}\lambda_{t}+\varepsilon_{it}$ （1）

Here, $y_{it}$denotes the score of person *i* at time t. $a_{i}$is intercept scores of person *i* for a variable, which represent the true scores at the first time point. $\beta_{i}$ is slope scores of person *i*, which represent the constant annual changes of true scores. $\lambda_{t}$is the value of the timing metric at time t in its original scale. $\varepsilon_{it}$ is residual at time t for person *i.*

If no differences in intercepts exist between individuals, then a common starting level exists for all individuals. If no differences in slope exist between individuals, then a common growth trajectory exists for all individuals. However, there is generally variation between individuals in both initial values and growth trajectories, which can then be explained by the following equation:

$$a_{i}=\mu_{\alpha}+\zeta_{\alpha i}$$

$$\beta_{i}=\mu_{\beta}+\zeta_{\beta i}$$

$\mu_{\alpha}$、$\mu_{\beta}$ denotes the mean of the intercept and slope of all individuals respectively. Then each individual has the same $\mu_{\alpha}$and$\mu_{\beta}$, so called fixed coefficients. $\zeta_{\alpha i}$ and$\zeta_{\beta i}$ indicate the differences in individual intercepts and slopes from $\mu_{\alpha}$ and $\mu_{\beta}$, respectively. Then each individual has a specific value, so called random coefficients.

Then equation (1) can be written as $y_{it}=\left( \mu_{\alpha}+\lambda_{t}\mu_{\beta} \right)+\left( \zeta_{\alpha i}+\lambda_{t}\zeta_{\beta i}+\varepsilon_{it} \right)$.

To sum up, the trajectory of change by the latent growth model was represented by two latent variables: a potential intercept growth factor $\alpha$, and a potential slope growth factor $\beta$. Specifically, the mean of the intercept factor (corresponding to $\mu_{\alpha}$) represents the average initial level and its variance (corresponding to $\zeta_{\alpha i}$) indicates the degree of variation in the initial level between individuals. The mean of the slope factor (corresponding to $\mu_{\beta}$) represents the average growth rate over the survey time, while its variance (corresponding to $\zeta_{\beta i}$) reflects the magnitude of inter-individual variation in growth rates.

**REFERENCES**

Grimm, K. J., & Ram, N. (2018). Latent Growth and Dynamic Structural Equation Models. *Annu Rev Clin Psychol, 14*, 55-89. doi:10.1146/annurev-clinpsy-050817-084840

Mengcheng, W., & Xiangyang, B. (2018). *Latent variable modelling and Mplus application - advanced*: Chongqing University Press.
