# Supplementary material for: Joint developmental trajectories and temporal precedence of physical function decline and cognitive deterioration: A longitudinal population-based study
Source: Front Psychol. 2022 Oct 12;13:933886. doi: 10.3389/fpsyg.2022.933886 (PMC9597508; doi:10.3389/fpsyg.2022.933886)
Supplement: Supplementary file 3 [file Image_1.pdf]

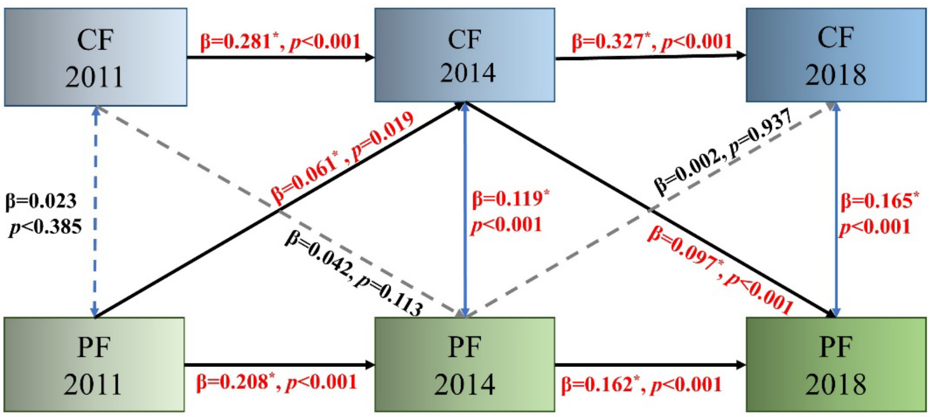

Figure S1. Autoregressive cross-lagged model controlling for covariates to assess bidirectional relationships between physical function and cognition over 3 years controlling covariates. Solid lines indicate significant associations, and dashed lines indicate non-significant associations. PF, physical function; CF, cognitive function, *p < 0.05.
